# Effects of cytomegalovirus infection on extravillous trophoblast cells invasion and immune function of NK cells at the maternal‐fetal interface

**DOI:** 10.1111/jcmm.15638

**Published:** 2020-09-07

**Authors:** Xiaoqian Lin, Yusha Chen, Zhuanji Fang, Qingshan Chen, Lichun Chen, Qing Han, Jianying Yan

**Affiliations:** ^1^ Department of Obstetrics Fujian Maternity and Child Health Hospital Affiliated Hospital of Fujian Medical University Fuzhou China; ^2^ Fujian Key Laboratory of Women and Children's Critical Diseases Research, Fujian Maternity and Child Health Hospital, Fujian Women and Children’s Hospital Fuzhou China; ^3^ Cervical Disease Diagnosis and Treatment Health Center Fujian Maternity and Child Health Hospital Affiliated Hospital of Fujian Medical University Fuzhou China

**Keywords:** cytomegalovirus, extravillous trophoblast, maternal‐fetal immune, proliferation

## Abstract

Cytomegalovirus (CMV) is one of the most common intrauterine infection virus, which can cause intrauterine transmission through the placenta, resulting in abortion, stillbirth and congenital malformations. In this study, the co‐culture extravillous trophoblast (EVT) HTR8/SVneo cell model of CMV infection was established in vitro. The toxicity of CMV infected EVT was determined, and then, the cell invasion experiment was conducted to evaluate the effect on the invasion ability of EVT cell lines. Western blot and real‐time PCR were used to detect the related cytokines in the PI3K/AKT signalling pathway in cells. Flow cytometry was used to detect the immune function related factors of the supernatant of CMV culture on decidual NK cells. The TCID50 of CMV virus was 10^−5.4^. The results of immunofluorescence showed that a large number of fluorescent green of CMV pp65 antigen signals appeared in the cytoplasm of CMV infection group. CMV could infect and replicate EVT cells and inhibited cell proliferation. The expression of proteins PDK1, AKT‐S473 and AKT‐S308 was significantly increased in CMV infection group. The levels of IL‐17, IL‐4 and IFN‐γ were 8.7 ± 0.48%, 12.17 ± 0.61% and 6.66 ± 0.25%, respectively, in CMV infection group. The above results indicated that CMV infection inhibited EVT cells proliferation, weakened the invasion ability and inhibited the immune function of NK cells at the maternal‐fetal interface, resulting in the abnormal maternal‐fetal crosstalk.

## 1 INTRODUCTION

Cytomegalovirus (CMV) is a linear double‐stranded virus, belongs to the subfamily blisters and has a high species specificity. It is one of the most common intrauterine infection viruses.^1^ It can cause intrauterine transmission through the placenta, causing abortion, stillbirth, intrauterine growth retardation and central nervous system injury in neonates.^2^ According to statistics, the infected rate of CMV in live births in the world was about 0.2% ~ 6.1%.^1^ The process and outcome of intrauterine transmission of CMV are related to the immune regulation at the maternal‐fetal interface.^3^ Placenta is located at the maternal‐fetal interface. Although its immune defence function is not perfect, it is still of great significance in preventing intrauterine transmission of pathogens.

Physiological pregnancy can induce partial immune suppression at maternal‐fetal interface. However, under the stimulation of inflammation, viral infection and other factors, the maternal‐fetal interface is damaged, which leads to the cloning activation of T and B cells of specific immune response and produces specific immune effect.^4^ It was found that extravillous trophoblast (EVT) had the ability of antigen presentation, which could effectively activate memory T cells and stationary T cells and improve the body's immunity.^5^, ^6^ At the same time, EVT cells can be infected with CMV in vivo and in vitro.^7^, ^8^ When CMV infection occurs at the maternal‐fetal interface, the EVT may present the antigen of CMV to the T cells infiltrating into the local area of inflammation and initiate a specific immune response. Therefore, we established the co‐culture cell model of CMV infected EVT in vitro and observed the effects of CMV infection on EVT invasion ability and immune function of NK cells at the maternal‐fetal interface from the PI3K/AKT signalling pathway.

## 2 MATERIALS AND METHODS

### 2.1 Materials

The HTR8/SVneo cells, derived from human first trimester EVT, were kindly provided by Charles Graham, Queen’s University, Kingston, Ontario, Canada; the CMV AD169 strain was provided by the Hubei Virus Research Institute; all peripheral blood samples were collected from pre‐pregnancy counselling healthy women in the Department of Obstetrics in Fujian Maternity and Child Health Hospital, Affiliated Hospital of Fujian Medical University. 10 mL venous blood in the middle of the elbow was sterile extracted and heparin anticoagulation was used. Transwell chamber (8.0 μm) (Corning); DMEM, FBS, penicillin, streptomycin, Trypsin (Gibco); RIPA, heparin (Sigma); PMSF (Amresco); SDS‐PAGE (Beyotime Biotechnology). Inverted microscope, fluorescence microscope (Olympus); Metash UV‐5100B spectrometer (Thermo).

This study was approved by the Ethics Committee of Fujian Maternity and Child Health Hospital, Affiliated Hospital of Fujian Medical University. Written informed consent was obtained from each woman.

### 2.2 Methods

#### 2.2.1 Virus amplification

This is a original research paper and the details of procedure as follows^9^: HTR‐8/SV Neo cells were cultured in high‐glucose DMEM medium containing 10% FBS, and CMV AD169 was cultured in high‐glucose DMEM medium containing 3% FBS. After 7 days, cells and culture medium were collected, frozen and thawing repeatedly for three times before centrifugation. Supernatant was taken to obtain the amplified virus venom, which was stored at −70°C.

#### 2.2.2 CMV toxicity measurement

After HTR‐8/SV neo cells grew into monolayer, the culture medium of each well was discarded. The diluted virus (×10) 0.1 mL was inoculated into the 96‐well cell culture plate. Normal cell control wells were set at each dilution degree. After discarding the venom or diluent, 1.0 mL cell culture medium was added to maintain the growth of cell in each well at 37°C for 24 hand then observed and recorded the results according to the following formula. The amount of 50% tissue cell infection was calculated (TCID50).

TCID50 = logarithm of the reciprocal of the high critical dilution of 50% infection + logarithm of the distance ratio × dilution factor. Where: distance ratio = (50% critical infection rate−50%)/(50% critical infection rate−50% low critical infection rate).

#### 2.2.3 The CMV infection model establishment of HTR‐8/SV neo cells

HTR‐8/SV neo cells were cultured in a 6‐well plate with high‐glucose DMEM medium containing 10% FBS for passage. After the supernatant was discarded overnight, culture medium was changed of high‐glucose DMEM containing 3% FBS. HTR‐8/SV neo cells were divided into control group and CMV infection group. Control group was added 100 μL PBS in each well, while the CMV infection group was added 100 μL TCID50 CMV AD169 in each well. After adsorption for 2 hours, PBS was washed for two times, and the cells were cultured with DMEM medium containing 3% FBS in 5% CO_2_ incubator for 48 hours. CMV AD169 induced HTR‐8/SV neo cells lesion was observed under inverted microscope.

#### 2.2.4 CMV pp65 antigen was detected by immunofluorescence chemistry

HTR‐8/SV Neo cells were cultured for 48 hours and were fixed at room temperature for 30 minutes with 4% polyformaldehyde. Mouse anti‐CMV pp65 monoclonal antibody (dilution: 1:1000) was added and incubated overnight at 4°C. Cy3‐labelled sheep antimouse IgG was added and incubated at 37°C for 1 hour. PBS washed for three times and observed under inverted fluorescence microscope.

#### 2.2.5 Proliferation migration of HTR‐8/SV Neo cells

All cell culture reagents and transwell chamber were incubated in a 37°C thermostatic water bath. Cells in logarithmic growth stage were collected and suspended in serum‐free medium. After counting, the concentration was adjusted to 1 × 10^5^/mL. 600 μL culture medium containing 20% FBS was added into lower chamber (bottom of 24‐well plate). 150 μL cell suspension was added to the upper chamber, and the culture continued for 24 hours under the condition of 37°C and 5% CO_2_. Carefully remove the chamber with tweezers, drain the upper chamber liquid and fix the methanol at room temperature for 30 minutes. The chamber was removed, and the upper chamber fixative fluid was sucked dry. The crystal violet dye was dyed at room temperature for 15‐30 minutes and washed with PBS for several times. The chamber was removed, and the upper chamber fluid was sucked. The cells on the membrane surface at the bottom of the upper chamber were carefully wiped with a wet cotton swab. Five random fields were counted under the microscope, and the results were calculated.

#### 2.2.6 Western blot analysis of PDK1, AKT‐S473 and AKT‐S308 expression

Total proteins of tissue were extracted from rat, and 20 μg proteins were sampled. 5% concentrated gel and 12% isolated gel were prepared, respectively, to isolate proteins by SDS‐PAGE. Objective and internal reference proteins were transferred to NC membrane and then closed with 5% skimmed milk powder sealing fluid for 2 hours at room temperature. Rabbit anti‐human primary antibody PDK1 (1:1000), rat anti‐human primary antibody AKT‐S473 (1:500), rat anti‐human primary antibody AKT‐S308 (1:1000) and rat anti‐human primary antibody β‐actin (1:1000) were added and incubated at 4°C overnight. TBST washed 4 times; then, HRP‐labelled sheep anti‐rat secondary antibody (1:5, 000) was added and incubated at 37°C for 1 hour. TBST washed four times. Colour was developed with ECL luminescent solution, protein bands were exposed by gel image analysis system, and images were photographed and quantitatively analysed. The experiment was repeated three times.

#### 2.2.7 Expression of AKT, PI3K and PDK1 mRNA in PI3K/AKT signalling pathway

The operation is carried out according to the instructions as following: the method of TRIzol was used to extract whole RNA. Nucleic acid protein complex was isolated by RIzolRNA agent. Nucleic acid protein complex was extracted by chloroform and precipitated in isopropanol. The complex was cleaned by 75% ethanol and purified by RNase‐free water. The expression level of GAPDH was selected as internal reference and reversed total RNA according an iScriptcDNA Synthesis Kit (Bio‐Rad Laboratories). Primers for PCR detection were designed and synthesized according to the information of target gene sequences, as shown in Table 1. The amplifications were performed in a 96‐well plate at 95°C for 10 minutes, followed by 40 cycles of 95°C for 15 seconds and 60°C for 1 minutes. Each sample was run in triplicate. The relative mRNAs expression was expressed using the 2−ΔΔCt method.

**Table 1:** Primer sequence list

Primer name	Primer sequence	Primer size (bp)
GAPDH‐F	GATGCCCCCATGTTCGTCAT	181
GAPDH‐R	TCTTCTGGGTGGCAGTGATG	
AKT‐F	TCCTCCTCAAGAATGATGGCA	181
AKT‐R	GTGCGTTCGATGACAGTGGT	
PI3K‐F	CCACGACCATCATCAGGTGAA	112
PI3K‐R	CCTCACGGAGGCATTCTAAAGT	
PDK1‐F	CTGTGATACGGATCAGAAACCG	191
PDK1‐R	TCCACCAAACAATAAAGAGTGCT	

#### 2.2.8 Effect of CMV infection on NK cytokines

Magnetic activated cell sorting system were used to isolate peripheral blood NK cells and decidual NK cells from normal pregnant women and collect decidual mononuclear cells. NK cells were inoculated in 96‐well plates and cultured for 3 days. NK cells and supernatant were collected for subsequent detection. Groups were divided as the following: CMV infection group: TCM was infected CMV for 72 hours; TCM control group: normal TCM without infecting; Control group: only NK cell growth fluid. FITC‐anti‐IFN‐γ, PE‐anti‐IL‐17 and APC‐anti‐IL‐4 were added and incubated at 4°C for 30 minutes; then, flow cytometry was used to detect the contents of NK cell surface molecules and cytokines.

### 2.3 Statistical analysis

All experiments were replicated independently at least three times. The data were analysed using one‐way analysis of variance (ANOVA) and are presented as the mean ± standard deviation (SD). Statistical significance was defined as *P* < .05.

### 2.4 Data and resource availability

All data generated or analysed during this study are included here in the published article. No applicable resources were generated or analysed during the current study.

## 3 RESULTS

### 3.1 CMV toxicity measurement

After infection of CMV infection on HTR‐8/SV neo cells for 72 hours, 50% critical infection rate of 70 and 50% low critical infection rate of 30 were recorded. According to the formula, TCID50 = lg(1/10−5)+(70−50)/(70−20) × lg10 = 5.4. The TCID50 of CMV infection HTR‐8/SV neo cells was 10^−5.4^.

### 3.2 The CMV infection model establishment of HTR‐8/SV neo cells

**FIGURE 1:**
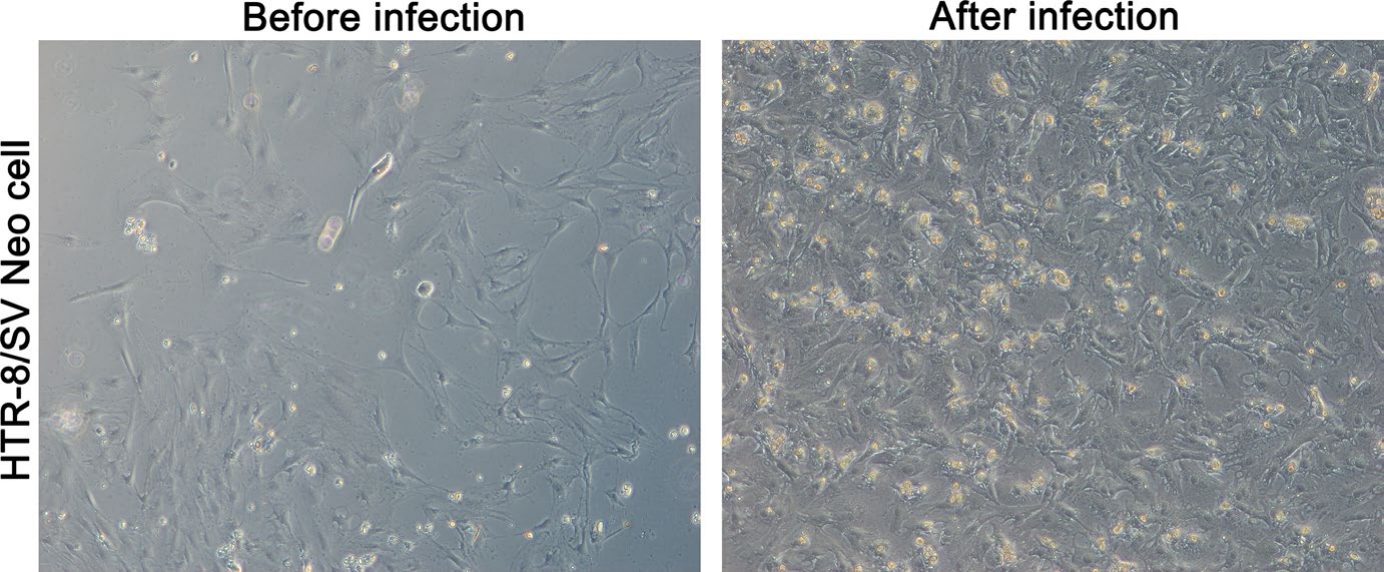
Microscope images of HTR‐8/SV Neo cells were infected by CMV. left, before infection; right, after infection

After CMV AD169 infecting HTR‐8/SV Neo cells, it was found that the cells were in normal state before infection, with fewer dead cells. After CMV AD169 infection, the number of cell death increased, as shown in Figure 1. Under the inverted microscope, the morphology of normal cells was fusiform, organelles were intact, intercellular boundaries were clear, and cells showed good monolayer growth. After infection, the cytopathic changes gradually became obvious with the extension of time, such as cell enlargement, swelling, gap widening, dimpling, serious organelle destruction, cell gap enlargement, and exfoliated cells and cell fragments in the culture medium.

### 3.3 CMV pp65 antigen was detected by immunofluorescence chemistry

**FIGURE 2:**
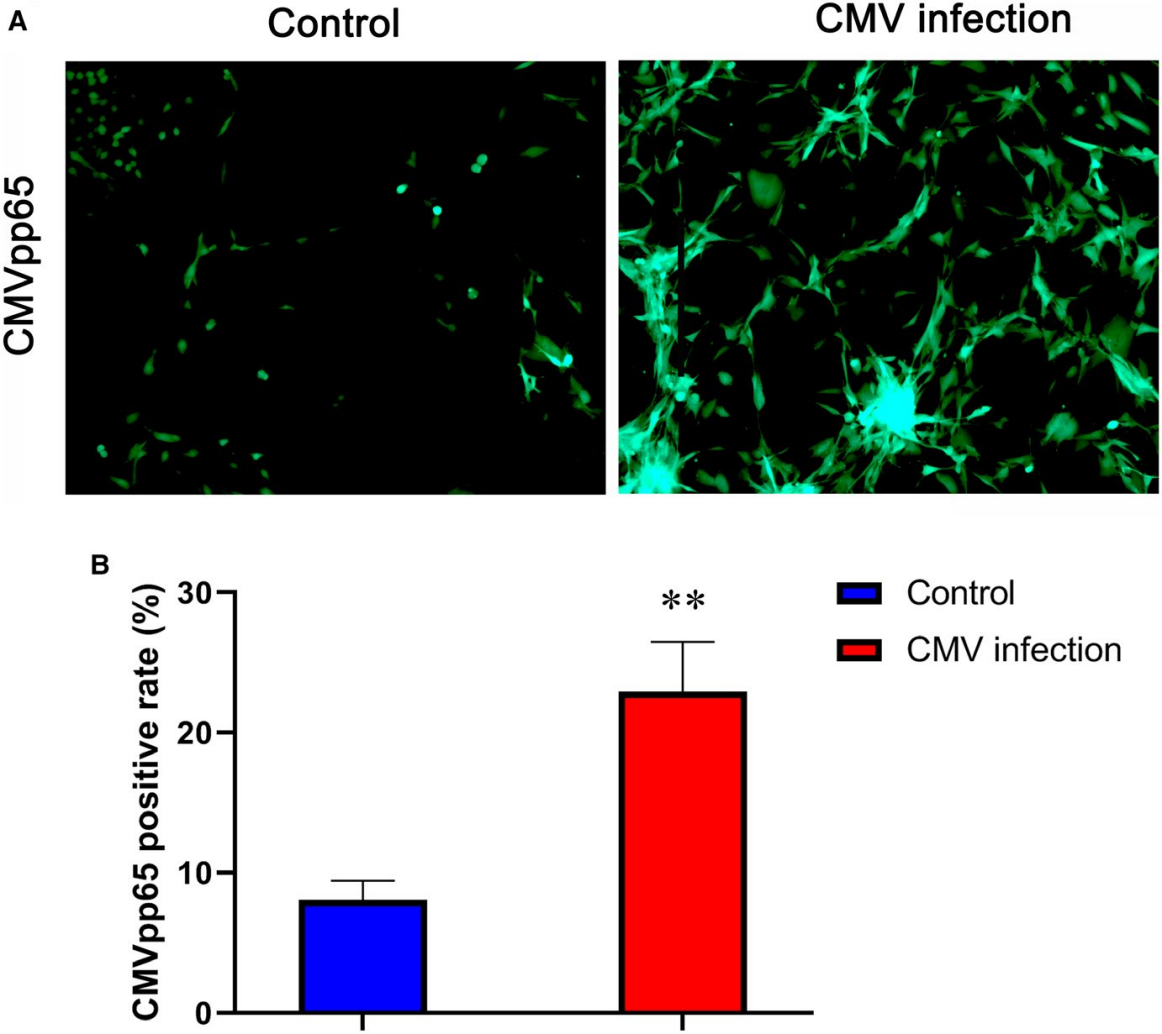
CMV pp65 antigen was detected by immunofluorescence chemistry. A, Fluorescent images, left: control group, right: CMV infection group (×200), fluorescent green signals were CMV pp65 antigen. B, The CMV pp65 positive rate of control group and CMV infection group, there was a significant difference. **P < .01 compared with control group. Data are shown as mean ± SD (n = 6 independent experiments)

The results of immunofluorescence chemistry showed that a large number of fluorescent green CMV pp65 antigen signals appeared in the cytoplasm of HTR‐8/SV Neo cells of CMV infection group, while no CMV pp65 antigen signal in the control group (Figure 2A). Moreover, the fluorescence brightness of CMV infection group was significantly higher than that of the Control group. This suggested that the model of HTR‐8/SV Neo cells infected with CMV was successfully established. The CMVpp65 positive rate in the CMV infection group was 22.9 ± 3.6%, much higher than that in Control group (8.0 ± 1.4%), as shown in Figure 2B. There was a significant difference (*P*＜.01).

### 3.4 Proliferation migration of HTR‐8/SV Neo cells

**FIGURE 3:**
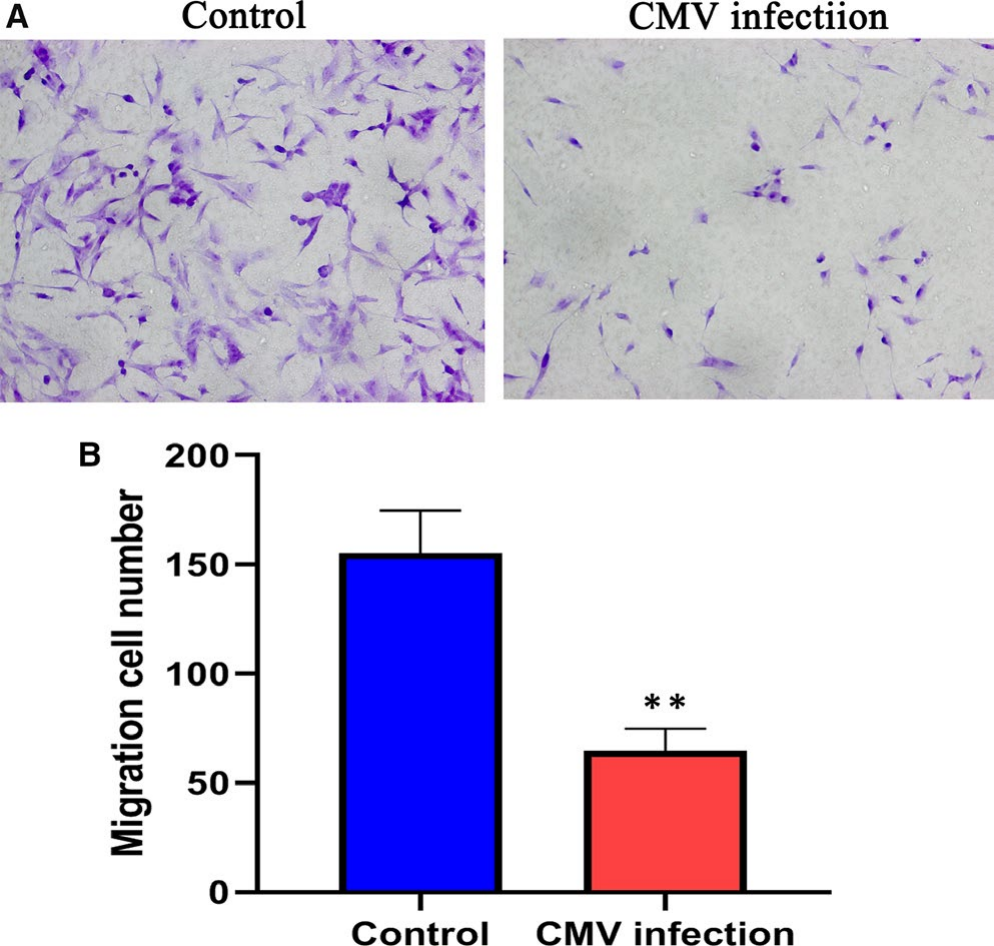
A, Microscopic image of HTR‐8/SV Neo cells, left: control group; right: CMV infection group. B, Migration cell number of control group and CMV infection group, there was a significant difference. **P < .01 compared with control group. Data are shown as mean ± SD (n = 6 independent experiments)

Cell migration refers to the movement of cells after receiving a migration signal or sensing a concentration gradient of certain substances. Compared with the control group, the number of cell migration in the CMV infection group was significantly reduced. The stained cells were shown in blue, as shown in Figure 3A. The statistical analysis results were shown in Figure 3B. The average number of cells migrated in the control group was 64 ± 10. The CMV infection group was 155 ± 20, and the control group was significantly higher than the CMV infection group, with statistically significant differences (*P* < .01). The disease venom can significantly inhibit the proliferation of HTR‐8/SV Neo cells within 24 hours, and CMV can infect and replicate in cells to inhibit the proliferation of cells.

### 3.5 Western blot analysis of PDK1, AKT‐S473 and AKT‐S308 expression

**FIGURE 4:**
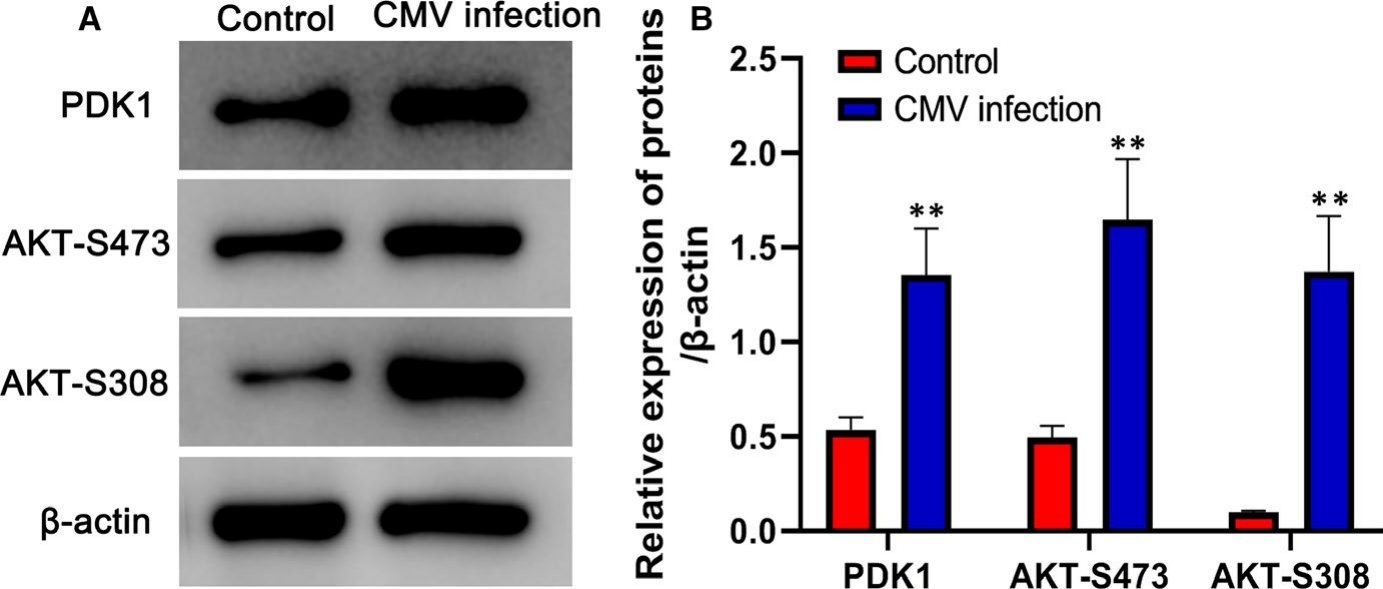
Protein levels of PDK1, AKT‐S473 and AKT‐S308 in different groups determined by Western blotting. A, Grey level images of different protein in control group and CMV infection. B, Relative expression of proteins of PDK1, AKT‐S473 and AKT‐S308. **P < .01 compared with control group. Data are shown as mean ± SD (n = 3 independent experiments)

The expression of proteins PDK1, AKT‐S473, AKT‐S308 was detected by Western Blot. As shown in Figure 4, proteins PDK1, AKT‐S473 and AKT‐S308 all significantly increased in CMV infection group compared with the control group. There was a significant difference (*P* < .01).

### 3.6 Expression of AKT, PI3K and PDK1 mRNA in PI3K/AKT signalling pathway

**FIGURE 5:**
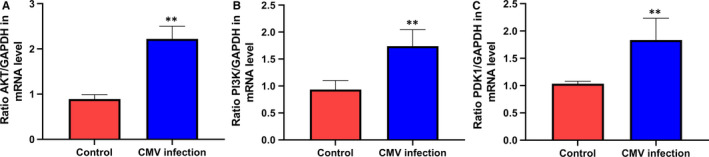
Relative levels of AKT, PI3K and PDK1 mRNA expression. A, the ratio of AKT/GAPDH; B, the ratio of PI3K/GAPDH; C, the ratio of PDK1/GAPDH. **P < .01 compared with control group. Data are shown as mean ± SD (n = 6 independent experiments)

The expression of AKT, PI3K and PDK1 mRNA in CMV infection group was obviously higher than that of control group. There was a significant difference (*P* < .01) (Figure 5).

### 3.7 Effect of CMV infection on NK cytokines

**FIGURE 6:**
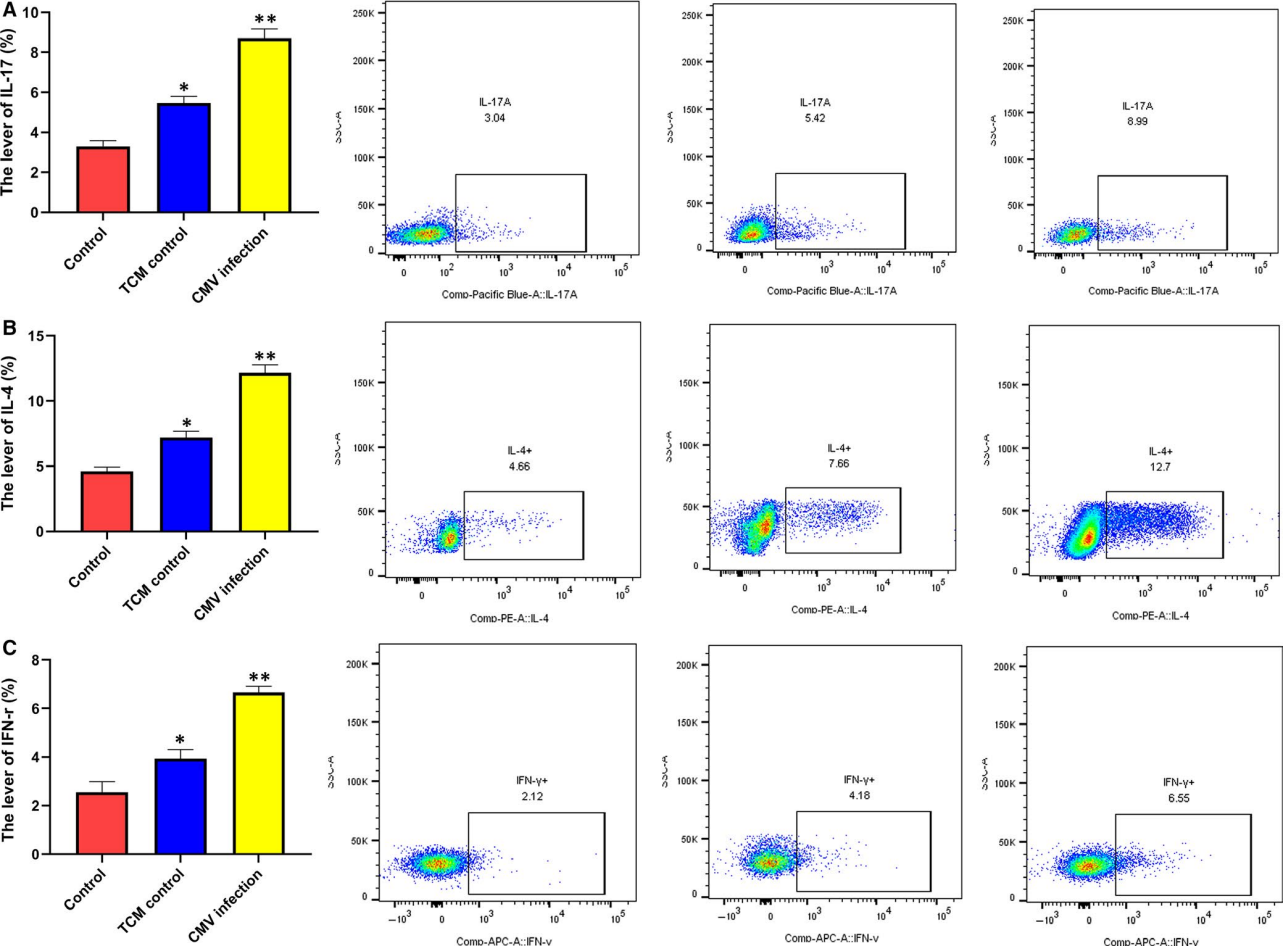
The expression of cytokines in each group was analysed by flow analysis. A, The level of IL‐17; B: the level of IL‐4; C: the level of IFN‐γ. *P < .05, **P < .01 compared with control group. Data are shown as mean ± SD (n = 6 independent experiments)

The levels of IL‐17, IL‐4 and IFN‐γ were 8.7 ± 0.48%, 12.17 ± 0.61% and 6.66 ± 0.25%, respectively, in CMV infection group. There is a significant difference, *P*＜0.05 in comparison with TCM group (5.47 ± 0.32%, 7.20 ± 0.48%, 3.94 ± 0.37%). *P*＜0.01 in comparison with control group (3.31 ± 0.28, 2.96 ± 2.52%, 1.56 ± 1.34%) (Figure 6).

## 4 DISCUSSION

CMV is a frequent cause of intrauterine infection, occurring in 0.2%–2.5% of live births in developed countries and 0.6%–6.1% in developing countries.^10^ CMV pp65 was one of the most important envelope proteins produced at the early stage of CMV replication, which promoted virus replication by regulating CMV gene expression and inhibited host cell metabolism.^11^ Pregnancy infection with CMV could cause miscarriage, stillbirth, intrauterine growth retardation, etc. During normal pregnancy, EVT plays an important role in embryo implantation and maternal‐fetal immune tolerance. Through proliferation, migration and invasion of the uterine decidua and superficial muscle layer, EVT carries out physiological reconstruction of the spiral uterine artery, ensuring the normal blood supply to the embryo or foetus.^12^ Fisher et al ^13^ reported that EVT is the main route of intrauterine infection of CMV. In this study, it was found that CMV could infect the EVT HTR‐8/SV Neo cells, and there were a large number of CMV pp65 antigen signals in the cells, which were duplicated in the cells, leading to the decline of cell proliferation, poor uterine spiral artery reconstruction, insufficient blood supply of the placenta, and abortion, stillbirth and intrauterine growth delay.^14^, ^15^

EVT was the main constituent cells of the placenta at the mother‐foetus interface, which could regulate the immune active cells of the mother's decidua and participate in maternal‐fetal immune tolerance through direct contact with the decidual membrane, secretion of cytokines.^16^ Once the immune crosstalk disorder at the maternal‐fetal interface appeared, it would lead to abortion and other pathological pregnancy.^17^ In this study, a CMV infection HTR‐8/SV Neo cell model was established in vitro. Then, the immune state of NK cells at the maternal‐fetal interface during CMV infection was simulated, and related cytokines were detected. NK cells could release a variety of cytokines, especially IFN‐γ, which was conducive to the differentiation of Th1 and the inhibition of Th2 by secreting IFN‐γ, thus affecting the acquired immune system. IFN‐γ was mainly secreted by Th1 cells, and IL‐4 and IL‐17 were mainly secreted by Th2 cells. These factors played an immunosuppressive role in immune response and were one of the main regulatory mechanisms to maintain immune tolerance.^18^, ^19^ Researchers have found that over expression of AKT promoted the expression of IFN‐γ, IL‐4 and IL‐17 in CMV infection HTR‐8/SV Neo cells, suggesting that the mechanism might target AKT to promote the PI3K/ AKT signalling pathway. The PI3K/AKT signalling pathway could regulate cell cycle, cell growth and cell proliferation and was closely related to cell differentiation.^20^, ^21^ It was also involved in the differentiation of immune cells and played a regulatory role in development and functional activity.

## 5 CONCLUSION

Cytomegalovirus infection inhibited the proliferation of extravillous cytotrophoblast (EVT) cells, weakened the invasion ability and inhibited the immune function of NK cells at the maternal‐fetal interface, resulting in the abnormal maternal‐fetal crosstalk.

## CONFLICT OF INTEREST

The authors have declared no conflict of interest.

## AUTHOR CONTRIBUTION

JY and XL were responsible for conception and design of the study. YC, ZF, LC and QH participated in the collection of data and samples. XL and QC performed the laboratory tests. XL and YC did the statistical analysis and wrote the manuscript. All authors approved the final manuscript.

## Data Availability

Data were available on request from the corresponding author.

## References

[1] Kabani N , Ross SA . Congenital cytomegalovirus infection. J Infect Dis. 2020;221(Supplement_1):S9–S14. 32134480 10.1093/infdis/jiz446 PMC8453618

[2] Lin X , Wang J , Wang Z , et al. Rare detection of cytomegalovirus in severe fetal malformations in China. J Clin Virol. 2016;79:54–60. 27089031 10.1016/j.jcv.2016.04.002

[3] Jabrane‐Ferrat N . Features of human decidual NK cells in healthy pregnancy and during viral infection. Front Immunol. 2019;10:1397. 31379803 10.3389/fimmu.2019.01397 PMC6660262

[4] Mor G , Aldo P , Alvero AB . The unique immunological and microbial aspects of pregnancy. Nat Rev Immunol. 2017;17 (8):469–482. 28627518 10.1038/nri.2017.64

[5] Du M‐R , Guo P‐F , Piao H‐L , et al. Embryonic trophoblasts induce decidual regulatory T cell differentiation and maternal–fetal tolerance through thymic stromal lymphopoietin instructing dendritic cells. J Immunol. 2014;192(4):1502–1511. 24453244 10.4049/jimmunol.1203425 PMC3918863

[6] Huang Y , Zhu X‐Y , Du M‐R , Li D‐J . Human trophoblasts recruited T lymphocytes and monocytes into decidua by secretion of chemokine CXCL16 and interaction with CXCR6 in the first‐trimester pregnancy. J Immunol. 2008;180(4):2367–2375. 18250446 10.4049/jimmunol.180.4.2367

[7] Tabata T , Petitt M , Fang‐Hoover J , Pereira L . Survey of cellular immune responses to human cytomegalovirus infection in the microenvironment of the uterine–placental interface. Med Microbiol Immunol. 2019;208(3‐4):475–485. 31065796 10.1007/s00430-019-00613-w PMC6635015

[8] Tabata T , Petitt M , Zydek M , et al. Human cytomegalovirus infection interferes with the maintenance and differentiation of trophoblast progenitor cells of the human placenta. J Virol. 2015;89(9):5134–5147. 25741001 10.1128/JVI.03674-14 PMC4403461

[9] Liu T , Zheng X , Chen J , et al. Effect of human cytomegalovirus on invasive capability of early pregnant extravillous cytotrophoblasts. J Huazhong Univ Sci Technolog Med Sci. 2011;31(6):819–823. 22173505 10.1007/s11596-011-0683-x

[10] Emery VC , Lazzarotto T . Cytomegalovirus in pregnancy and the neonate. F1000Res. 2017;6:138. 28299191 10.12688/f1000research.10276.1 PMC5310379

[11] Cristea IM , Moorman NJ , Terhune SS , et al. Human cytomegaloviruspUL83 stimulates activity of the viral immediate‐early promoter throughits interaction with the cellular IFI16 protein. J Virol. 2010;84:7803‐7814. 20504932 10.1128/JVI.00139-10 PMC2897612

[12] Liu T , Zheng X , Li Q , Chen J . Role of human cytomegalovirus in the proliferation and invasion of extravillous cytotrophoblasts isolated from early placentae. Int J Clin Exp Med. 2017;8:17248‐17260. PMC4694217 26770317

[13] Fisher S , Genbacev O , Maidji E , Pereira L . Human cytomegalovirus infection of placental cytotrophoblasts in vitro and in utero: implications for transmission and pathogenesis. J Virol. 2000;74:6808‐6820. 10888620 10.1128/jvi.74.15.6808-6820.2000 PMC112198

[14] James JL , Stone PR , Chamley LW . The effects of oxygen concentration and gestational age on extravillous trophoblast outgrowth in a human first trimester villous explant model. Hum Reprod. 2006;21:2699‐2705. 16807282 10.1093/humrep/del212

[15] Zhang L , Men K , Zhang JX , et al. Establishment and biological characterization of an Asian human first trimester placental villus‐derived trophoblastic cell line (HPT‐8) in Vitro. Pract J Obstet Gynecol. 2006;22:232‐236.

[16] Yao Y , Song J , Wang W , Liu N . Decidual vascular endothelial cells promote maternal–fetal immune tolerance by inducing regulatory T cells through canonical Notch1 signaling. Immunol Cell Biol. 2016;94:458‐469. 26714886 10.1038/icb.2015.119

[17] Adams Waldorf KM , Gammill HS , Lucas J , et al. Dynamic changes in Fetal Microchimerism in maternal peripheral blood mononuclear cells, CD4+ and CD8+ cells in normal pregnancy. Placenta. 2010;31:589‐594. 20569981 10.1016/j.placenta.2010.04.013 PMC2923456

[18] Shimamura M , Murphy‐Ullrich JE , Britt WJ . Human cytomegalovirus induces TGF‐beta1 activation in renal tubular epithelial cells after epithelial‐to‐mesenchymal transition. PLoS Pathog. 2010;6:e1001170. 21079788 10.1371/journal.ppat.1001170 PMC2973835

[19] Jackson SE , Mason GM , Wills MR . Human cytomegalovirus immunity and immune evasion. Virus Res. 2011;157:151‐160. 21056604 10.1016/j.virusres.2010.10.031

[20] Luyendyk JP , Schabbauer GA , Tencati M , Holscher T , Pawlinski R , Mackman N . Genetic analysis of the role of the PI3K‐Akt pathway in lipopolysaccharide‐induced cytokine and tissue factor gene expression in monocytes/macrophages. J Immunol. 2008;180:4218‐4226. 18322234 10.4049/jimmunol.180.6.4218 PMC2834303

[21] Hazeki K , Nigorikawa K , Hazeki O . Role of phosphoinositide3‐kinase in innate immunity. Biol Pharm Bull. 2007;30:1617‐1623. 17827709 10.1248/bpb.30.1617

